# Who is the patient with resistant myofascial temporomandibular disorders pain? A somatosensory, psychosocial, and genetic characterization

**DOI:** 10.1186/s10194-025-02055-7

**Published:** 2025-05-06

**Authors:** Giancarlo De la Torre Canales, Rodrigo Lorenzi Poluha, Flávia Fonseca Carvalho Soares, Dyna Mara Araújo Oliveira Ferreira, Alfonso Sánchez-Ayala, Leonardo Rigoldi Bonjardim, Malin Ernberg, Paulo César Rodrigues Conti

**Affiliations:** 1 Department of Prosthodontics, Bauru Orofacial Pain Group, Bauru School of Dentistry, University of São Paulo, Sao Paulo, Brazil; 2 Department of Biological Sciences, Bauru Orofacial Pain Group, Bauru School of Dentistry, University of São Paulo, Sao Paulo, Brazil; 3 Division of Oral Rehabilitation, Department of Dental Medicine, Karolinska Institutet, Huddinge, Sweden; 4 Egas Moniz Center for Interdisciplinary Research (CiiEM), Egas Moniz School of Health & Science, Caparica, Almada, Portugal; 5 Department of Dentistry, State University of Maringá, Paraná, Brazil; 6 Department of Dentistry, University of Ponta Grossa, Ponta Grossa, Paraná Brazil

**Keywords:** Temporomandibular disorders, Myofascial pain, Refractory pain, Chronic pain, Phenotype

## Abstract

**Background:**

Resistance to treatments have been assessed in chronic conditions such as migraine, but not in temporomandibular disorders (TMD). This study aimed to identify factors that influence treatment outcome in patients with myofascial TMD pain.

**Methods:**

Seventy-two females were divided into three groups: TMD successfully treated (TMD-S, *n* = 24), TMD resistant to treatment (TMD-R, *n* = 24) and Controls without TMD (*n* = 24). Criteria for resistance included: less than 30% pain reduction after three months of conservative treatment and an average pain intensity > 50 mm (VAS) during the last month. Quantitative sensory testing (QST), psychosocial status and genetic polymorphisms were examined. ANOVA on ranks (psychosocial variables) with Dunn’s test as post-hoc or ANOVA (age and somatosensory variables) with Tukey test as post-hoc test, and Dwass-Steel-Critchlow-Fligner test (genetic variables) were used for univariate groups comparisons. Multivariate statistics were used to identify outcomes that separated the groups.

**Results:**

QST assessment revealed lower baseline pressure pain threshold and higher wind-up ratio in the trigeminally and spinally innervated areas in the TMD-R group compared with the other groups (*p* = 0.01). Also, the TMD-R group presented higher values in all assessed psychosocial variables (*p* < 0.01) and higher prevalence of the HTR1A polymorphism rs6295 (*p* = 0.02) compared with the other groups at baseline. Multivariate analysis showed that the three variables that distinguished the best between TMD-R and TMD-S were sleeping quality, central sensitization, and depressive symptoms.

**Conclusion:**

Psychosocial, somatosensory, and genetic alterations are related to unsuccessful treatment response in myofascial TMD patients.

## Introduction

Chronic primary pain is characterized by persistent or recurring pain lasting more than 3 months in one or more body areas. This pain is accompanied by notable emotional distress or functional impairment, and the symptoms are not attributable to any other diagnosis [1]. Its underlying mechanisms involve a spectrum of peripheral and central processes, with some of them potentially elucidated through the concept of nociplastic pain [2]. Therefore, chronic pain is an overarching category including the most prevalent and clinically significant chronic pain conditions, such as chronic primary headache and orofacial pain (eg, temporomandibular disorders, TMD) [3]. Furthermore, the prevalence of chronic pain is approximately 20% [4, 5]. Due to this prevalence, the economic costs to treat this condition are substantial, and could potentially reach as much as US$635 billion per year in medical cost and loss of productivity [6].

Even though several treatment guidelines have been published for chronic pain, treating this condition is challenging [7]. Treatment is symptom-based and multimodal, and customized to enhance quality of life and diminish pain levels [8]. However, sometimes treatment is not successful. The Pain Study Tracking Responses for a Year (PainSTORY) reported that among chronic pain patients dissatisfied with their pain management, 67% experienced no change and 11% had increased pain levels over 12 months [9]. Chronic pain that resists relief from multiple therapeutic interventions has been denominated as “refractory and resistant” which mainly differ in the time frame of lack of response to treatment, 3 months for resistant and 6 months for refractory as proposed in some classifications of chronic pain conditions [10, 11]. In a broader proposed definition, pain is refractory and resistant when multiple evidence-based therapies used in a clinically appropriate fashion failed to reach treatment goals and when psychiatric disorders and psychosocial factors that could influence pain outcomes have been assessed and addressed [12].

“Resistant pain” in TMD has not been properly addressed, mainly because the prevalence of patients needing significant clinical treatment is low (10%−15%) [13], and to the high percentage of positive responders to conservative (biopsychosocial) treatments [14]. However, treating chronic orofacial pain disorders like TMD is challenging because the trigeminal system involves complex neural networks connected to the sympathetic, parasympathetic, and cervical nervous systems [15, 16]. Moreover, one-half to two-thirds of TMD patients, still experience pain upon reevaluation after 6 months [17, 18]. This pinpoints the heightened risk of developing chronic pain, and the importance of selecting the most appropriate treatment at the beginning to avoid resistant/refractory pain.

Treatment difficulties augment when one considers the biopsychosocial nature of TMDs [19–21] and the genetic relation with chronic TMD [22]. Pain amplification, psychological profile as well as global symptoms are key factors in the etiology of painful TMDs [23, 24]. Additionally, the Orofacial Pain: Prospective Evaluation and Risk Assessment (OPPERA) study, reported single-nucleotide polymorphisms (SNPs) (*HTR2 A*/rs9316233, *NR3 C1*/rs2963155) that suggest a contribution of the hypothalamic–pituitary–adrenal system and the nociceptive and affective pathways to chronic TMD [22]. Considering all these factors may help to identified profiles (phenotype and genotype) of patients that respond or not to TMD treatment and will add valuable clinical insights, allowing clinicians to understand which factors influence successful TMD treatment outcomes.

This study investigated the prevalence of resistant myofascial TMD pain and identified somatosensory, psychosocial, and genetic factors affecting treatment outcomes and factors distinguishing between healthy controls and patients with myofascial TMD.

## Methods

### Settings and study design

This prospective observational study was conducted at the orofacial pain clinic at the Bauru School of Dentistry from October 2019 to May 2021. The study was approved by the Research Ethics Committee of the Bauru School of Dentistry, University of Sao Paulo, Brazil (CAAE # 79,079,917.6.0000.5417/CAAE # 82,201,818.3.0000.5417) and was conducted following the Helsinki Declaration. All subjects were informed about the research aims and voluntarily signed an informed consent form to participate in this study. Reporting of the data followed the recommendations of the Strengthening the Reporting of Observational Studies in Epidemiology (STROBE) guidelines.

### Subjects

The sample of this study was composed of 72 Brazilian females including TMD cases and healthy controls (*n* = 24). TMD cases were divided in two groups according to treatment response, after a three-months treatment protocol in: TMD cases with successful treatment (TMD-S, *n* = 24), TMD cases resistant to treatments (TMD-R, *n* = 24). For the TMD cases, consecutive patients with TMD of muscular origin treated at or referred to the orofacial pain clinic of the Bauru School of Dentistry, University of Sao Paulo were recruited. The general inclusion criteria for the TMD cases were females, aged over 18 years, with a diagnosis of myofascial pain (MFP) and MFP with referral, according to the Brazilian Portuguese version of the Diagnostic Criteria for TMD (DC/TMD) [25]. Patients in the TMD-S group, should have reported a pain reduction of more than 30% after three months of conservative treatment. Patients in the TMD-R group should have reported a pain reduction of less than 30% and an average pain intensity > 50 mm on a 0–100 mm Visual Analogue Scale (VAS) during the last month of the 3 months treatment period. Mandatory treatments for all patients included counselling (consisted in self-care strategies to control parafunctions and pain (thermotherapy and massage), information about the etiology and prognosis of TMD, sleep quality, dietary habits and relaxion techniques), oral appliance (flat occlusal appliance covering the superior teeth, made of rigid acrylic resin and adjusted to reach maximum bilateral contacts) which were applied for three months and pharmacotherapy (acting peripherally and centrally like, NSAIDs/10 days, muscle relaxants/1 month and antidepressive/2 months), while dry or wet needling, acupuncture, and physiotherapy were used as additional treatments. All patients received the mandatory treatments, but regarding pharmacotherapy, the doses were tailored for each patient accordingly to symptoms. The inclusion criteria for the control group were females, aged over 18 years and good general health.

The exclusion criteria for the three groups were presence of dental and neuropathic pain, traumas to the face and neck, uncontrolled systemic disorders, (e.g., diabetes), hypertension, or endocrine disorders; major psychiatric disorders, systemic inflammatory diseases (e.g., arthritis) presence of congenital or developmental changes, (e.g., aplasia, hyperplasia, dysplasia, or neoplasms), and ongoing orthodontic treatment and fibromyalgia. For the control group the exclusion criteria included presence of primary headache, TMD, fibromyalgia and other chronic painful syndromes.

### Outcomes

#### Pain variables

The characteristic pain intensity (CPI) was used to compare the participants pain intensity at baseline. This is composed of three 0–10 numerical rating scales (NRS) where 0 represent no pain and 10 represents the worst pain imaginable assessing the current pain intensity and the worst and average pain intensities during the last month. The CPI is the average of the three scores multiplied with 10, giving a score 0–100. For other pain assessments (see below) a 0–100 VAS with the same descriptors for 0 and 100 as for the NRS, was used [25].

#### Somatosensory profile

Three tests of the quantitative sensory test (QST) battery for mechanical somatosensory assessment were considered: (a) mechanical pain threshold (MPT), (b) wind up ratio (WUR), and (c) pressure pain threshold (PPT). In the TMD-S and TMD-R groups, measurements in the trigeminal area were performed in the masseter muscle on the side that the patient reported more severe pain, and in the control group on the dominant side (masseter insertion region). In the spinally innervated area, the same tests were performed at the hand on the same side as the most painful masseter in the TMD-S and TMD-R groups and at the dominant hand for the control group. Conditioned pain modulation (CPM) was used to assess endogenous pain inhibition.

### Mechanical pain threshold

For this test, a standardized set of Semmes–Weinstein monofilaments (Touch-Test TM Sensory Evaluators; North Coast Medical Inc, California, USA), which applies forces between 0.008 g/mm^2^ and 300 g/mm^2^ were used. The monofilaments were applied perpendicularly to the examination sites and a light pressure was applied until the filament curved. Participants were instructed to verbally report when feeling a “prick, pinprick, or slightly painful sting” sensation in the contact area of the monofilaments. The limit method was used to determine the threshold using a series of five ascending and five descending stimuli. The MPT was considered as the geometric mean of these repetitions [26, 27].

### Wind-up ratio

To measure WUR, the smallest Von Frey filament that caused a sensation of mild pain (30 to 50 on VAS) was used. The filament was placed on the skin over the assessed regions and pressure was applied until the filament curved. This test was performed in a continuous sequence where the intensity of a single painful stimulus was compared with that of a series of 10 consecutive stimuli with the same filament and with the same intensity of force (1 per second applied within an area 1 cm^2^). Single pinprick stimuli were alternated with a train of 10 stimuli until both were done three times. Pain intensity values were quantified (VAS) after the single stimulus and at the end of the series of 10 consecutive stimuli. The wind-up ratio was calculated as the mean score of the three series of 10 stimuli divided by the mean score of the three single stimuli [26, 27].

### Pressure pain threshold

Pressure pain threshold measurements were performed using a digital pressure algometer (model Kratos DDK-20, Sao Paulo, Brazil), with a flat circular tip of 1 cm^2^, through which a constant and increasing pressure of approximately 0.5 kg/cm2/s was applied. Participants were instructed to press a button connected to the algometer when the sensation of pressure turned into a slightly painful sensation to interrupt the exerted pressure. The recordings were repeated three times at 1-min intervals. The arithmetic mean of the three measurements in the sequence was calculated and used in analyses [26, 27].

### Conditioned pain modulation

To assess the CPM paradigm, as test stimulus (TS), PPT was first obtained in the masseter and thenar muscle, as explained above. As conditioning stimulus (CS), participants were asked to immerse the non-dominant hand in a container with water at 10 °C for 60 s. Then, a second assessment of PPT was immediately done at the end of CS in the same sites. In each evaluation, PPT was determined as the arithmetic mean of three measurements. The absolute (kgf/cm^2^) and percent (%) differences between PPT values (‘TS before CS’ and ‘TS after CS’) were considered as the CPM values. Negative values indicate an increase in pain threshold [28, 29].

### Psychosocial profile

The psychosocial profile was assessed using the validated Brazilian Portuguese translations of the following self-report questionnaires: Hospital Anxiety and Depression Scale (HADS) [30]; Central Sensitization Inventory (CSI) [31]; Perceived Stress Scale (PSS) [32]; Pittsburgh Sleep Quality Index (PSQI) [33], and Pain Catastrophizing Scale (PCS) [34].

### Hospital anxiety and depression scale

This questionnaire is composed of 14 multiple-choice questions (each scored 0–3), divided into two interleaved subscales, one for anxiety (7 questions) and another for depression (7 questions). The scores range from 0 to 21 points; the higher the value, the greater the symptoms of anxiety (A-HADS) and/or depression (D-HADS) [30].

### Central Sensitization inventory

This questionnaire is designed as an easy-to-administer screener for patients who are at high risk of having Central Sensitization or to assess Central Sensitization-related symptoms. It is divided in two parts: part A with 25 statements about current health symptoms and part B assessing previously diagnosed central sensitivity syndromes and related conditions. For part A, subjects mark the frequency in a scale ranging from 0–4 (0 = never and 5 = always) and after summing all the items, they are classified into five severity levels (subclinical, mild, moderate, severe, extreme) [31].

### Perceived stress scale

This questionnaire consists of 14 items that measures the degree of perceived stress in the last month considering the individual's global context. The scale total is the sum of the scores (0–4) for these 14 items and the scores can range from 0 to 56 [32].

### Pittsburgh sleep quality Index

This questionnaire consists of 19 self-report questions that assess various factors related to sleep quality, distinguishing individuals who sleep “well” or “poorly” and providing clinical information about different sleep disorders that can influence the sleep quality. The score ranges from 0 to > 10, and the higher the score (0–21), the worse the quality of sleep [33].

### Pain catastrophizing scale

This questionnaire measures catastrophic thoughts about pain. The subjects indicate the frequency of catastrophic thoughts when pain is severe. It consists of 13 statements in total in which the subjects mark the frequency in a scale ranging from 0–5 (0 = hardly ever and 4 = almost always). The total score is the sum of all the items ranging from 0 to 52 points. The higher the value, the more the degree of catastrophizing [34].

### DNA collection and single-nucleotide polymorphism analysis

Samples of 5 ml of unstimulated total saliva were collected in a 50 ml Falcon tube and storage at −80 degrees. Genomic DNA was extracted from epithelial buccal cells present in saliva using QIAmp DNA Mini Kit (Qiagen, Hilden, Germany), according to the manufacturer's instructions. The analysis of the genotypes of the selected polymorphisms HTR1B rs6296, HTR1 A rs6295, OPRM1 rs1799971, COMT rs4680, rs4633, and rs4818, and SCN9 A rs6746030) was performed using the Real-Time Polymerase Chain Reaction technique. The reactions were performed on the Viia 7 thermal cycler (Applied Biosystems, Massachusetts. USA), using the TaqMan method using TaqMan SNP genotyping assays (Applied Biosystems, Massachusetts. USA) pre-standardized and experimentally validated, following the manufacturer's instructions. All samples were analyzed at the end of the study after groups division.

All data collection was performed at baseline (before treatments).

### Statistical analysis

The sample size calculation was performed using G*Power 3.1.9.2 software and was based on calculated effect sizes. The following parameters were considered within a one-way ANOVA analysis: α error probability = 0.05, power (1-β error probability) = 0.8, number of groups = 3, and effect size values for PPT, CPM, MPT, and WUR of ≥ 0.4. The total sample size was calculated to be 66 subjects equally divided into three groups. However, considering an assumed withdrawal or dropout rate, 24 subjects were included in each group.

Univariate data were analyzed with SPSS. Data were assessed for distribution normality using the Shapiro–Wilk test. Normal distributions were found for age and somatosensory variables (MPT, WUR, PPT and CPM) that is why, comparisons between groups were done using ANOVA with Tukey test as post-hoc test. As the psychosocial variables (anxiety, depression, central sensitization, stress, sleep, and pain catastrophizing) are composed of scores obtained from individual ordinal scales, the sum scores cannot be regarded as continuous. Therefore, ANOVA on ranks (Kruskall-Wallis test) with Dunn’s test as post-hoc test was used to test for group differences. For the number of other painful TMD diagnosis (arthralgia and headache attributed to TMD) the Chi Square test was used. The Dwass-Steel-Critchlow-Fligner test was used to compare the proportion of polymorphisms between groups.

Since many of the outcomes are more or less interrelated, multivariate statistics were used to identify outcomes that separated the groups using SIMCA-P + v.17.0 (Sartorius Stedim Biotech, Umeå, Sweden) as earlier described [35]. Principle component analysis (PCA) was first used to investigate the correlation patterns for the investigated variables and to detect moderate or strong outliers among the observations using Hotelling’s T2 and DMod. No outliers were detected. Thereafter orthogonal partial least squares discriminant analysis (OPLS-DA) was used to regress group membership, i.e. multivariate correlations between variables and group membership. R2 describes the goodness of fit, i.e., the fraction of sum of squares of all the variables explained by a principal component whereas Q2 describes the goodness of prediction, i.e., the fraction of the total variation of the variables that can be predicted by a principal component using cross validation methods. R2 should not be considerably higher than Q2; if substantially higher (> 0.3) the robustness of the model is poor [36]. To validate the model, an obtained CV-ANOVA was used to test the significance of the model. The OPLS-DA model was considered significant if the CV-ANOVA had a *p*-value < 0.05. The variable influence on projection (VIP) value indicates the relevance of each X-variable pooled over all dimensions and the Y-variables indicate the group of variables that best explain Y. P(corr) was used to note the direction of the relationship (positive or negative). VIP > 1.0 and an absolute p(corr) > 0.4 was considered significant.

## Results

Out of the 226 myofascial pain patients who sought treatment at the orofacial pain clinic of the Bauru School of Dentistry during the study period, 10.6% (24) did not respond to the treatment protocols after three months, were considered resistant to treatment and allocated to the TMD-R group. The number of patients included in the other groups were randomly chosen (http://www.randomization.com/ in blocks of two patients) according to the number of patients included in the TMD-R. All patients were women of Latino ethnicity. The mean age (± standard deviation, SD) of the TMD-R group (44 ± 7 yrs) was significantly higher compared with TMD-S (38 ± 10 yrs) and control (32.2 ± 8.7 yrs) groups (*p* = 0.01). No other major differences in demographic characteristics were found across groups. No significant differences were found regarding the CPI of the TMD-R (7.4 ± 1.3) compared with the TMD-S (7.0 ± 1.5) (*p* > 0.05) at baseline, and no significant differences were found regarding pain duration between both groups (*p* > 0.05). The TMD-S group presented a higher presence of other painful TMD diagnoses (arthralgia and headache attributed to TMD) compared to the TMD-R group (*p* = 0.03).

### Somatosensory variables

The TMD-R group had lower PPTs and higher WURs values compared with the other groups (*p* = 0.01) in both the trigeminally and spinally innervated areas. The patient groups had lower MPTs than the controls in both the trigeminally and spinally innervated areas (*p* = 0.01), but there were no significant differences between the TMD-S and TMD-R groups. For CPM, no significant differences were found between groups for the trigeminally and spinally innervated areas (*p* > 0.05) (Table 1).

**Table 1:** Baseline somatosensory and conditioned pain modulation data assessed at the masseter and thenar muscles in patients with temporomandibular disorders pain resistant (TMD-R) or successful (TMD-S) to treatment, and in healthy controls. Data are presented as mean (± sd)

	TMD-R		TMD-S		Control	
	*n* = 24		*n* = 24		*n* = 24	
	Masseter	Thenar	Masseter	Thenar	Masseter	Thenar
PPT (kgf/cm^2^)	1.0 (± 0.4)*	2.5 (± 1.2)*	1.7 (± 1.0)	4.5 (± 1.5)	1.9 (± 0.9)	4.1 (± 1.0)
MPT (g/mm^2^)	10.6 (± 12.5)	53.2 (± 53.8)	8.0 (± 15.9)	15.6 (± 39.3)	123.6 (± 147.8)*	149.4 (± 169.4)*
WUR (0–100 VAS)	2.3 (± 1.2)*	2.2 (± 1.6)*	1.9 (± 0.7)	1.8 (± 0.5)	1.5 (± 0.6)	1.4 (± 0.4)
CPM (kgf/cm^2^)	−0.1 (± 0.1)	−0.3 (± 0.7)	−0.3 (± 0.7)	−0.3 (± 0.6)	−0.4 (0.7)	−0.7 (± 1.3)

*PPT* Pressure pain threshold, *MPT* Mechanical pain threshold, *CPM* Conditioned pain modulation, *WUR* Wind-up ratio

ANOVA test: * *p* <.001 between groups

### Psychosocial variables

Scores for all questionnaires are shown in Table 2. Inter-group comparison showed higher values for the TMD-R group when compared with the other groups for A- HADS and D-HADS (*p* = 0.01), CSI (*p* = 0.01), PSS (*p* = 0.03), PSQI (*p* = 0.01), PCS (*p* = 0.01). Furthermore, higher values were also found in the TMD-S group for CSI (*p* = 0.01), PCS (*p* = 0.01) and PSQI (*p* = 0.01) compared with the control group (Table 2).

**Table 2:** Baseline psychosocial data in patients with temporomandibular disorders pain resistant (TMD-R) or successful (TMD-S) to treatment, and in healthy controls. Data are presented as median (min–max)

	TMD-R	TMD-S	Control	*p*
	*n* = 24	*n* = 24	*n* = 24	
A-HADS *(total)*	8.0 (3-20)^*^	4.0 (0-19)	5.0 (1-13)	0.01
D-HADS *(total)*	8.0 (1-14)^*^	5.0 (0-10)	3.0 (0-8)	0.01
CSI *(total)*	50.0 (21-85)^*^	37.5 (12-80)^#^	25.0 (7-42)	0.01
PSS *(total)*	29.5 (13-40)^*^	21.0 (9-28)	21.50 (6-38)	0.03
PSQI *(total)*	10.0 (4-17)^*^	6.0 (0-14)^#^	4.0 (3-13)	0.01
PCS *(total)*	26.5 (10-49)^*^	19.5 (0-46)^#^	11.0 (0-23)	0.01

*HADS *Hospital Anxiety (A) and Depression (D) Scale, *CSI *Central Sensitization Inventory, *PSS *Perceived Stress Scale, *PSQI *Pittsburgh Sleep Quality Index, *PCS *Pain Catastrophizing Scale

Kruskall Wallis test (*p*) with Dunn’s test as postdoc: ^*^p<0.05: significant differences between TMD-R and the other groups. ^#^*p*<0.05: significant differences between TMD-S and Control group

### Genetic variables

The inter-group comparisons showed that the TMD-R group presented a higher prevalence of the polymorphic genotype for HTR1 A rs6295 when compared with the other groups (*p* = 0.02). Likewise, the TMD-R group presented a higher prevalence of the polymorphic genotype for HTR1B rs6296 compared to the control group (*p* = 0.01). No significant group differences were found for the other genotypes (Table 3).

**Table 3:** Genotype frequency (%) for each SNP in patients with temporomandibular disorders pain resistant (TMD-R) or successful to treatment (TMD-S), and in healthy controls

	TMD-R		TMD-S		Control		*p*
	*n* = 24		*n* = 24		*n* = 24		
Genotype	Ancestral	Polymorphic	Ancestral	Polymorphic	Ancestral	Polymorphic	
COMT rs4680	GG	GA + AA	GG	GA + AA	GG	GA + AA	
*n (%)*	10 (41.7)	14 (58.3)	12 (50.0)	12 (50.0)	8 (33.3)	16 (66.7)	0.8
COMT rs4818	GG	GC + CC	GG	GC + CC	GG	GC + CC	
*n (%)*	4 (16.7)	20 (83.3)	10 (41.7)	14 (58.3)	3 (12.5)	21 (87.5)	0.9
COMT rs4633	CC	CT + TT	CC	CT + TT	CC	CT + TT	
*n (%)*	10 (41.7)	14 (58.3)	12 (50.0)	12 (50.0)	8 (33.3)	16 (66.7)	0.8
HTR1 A rs6295	GG	GC + CC	GG	GC + CC	GG	GC + CC	
*n (%)*	2 (8.3)	22 (91.7)	10 (41.4)	14 (58.3)	11 (45.8)	13 (54.2)	0.02*
HTR1B rs6296	CC	CG + GG	CC	CG + GG	CC	CG + GG	
*n (%)*	9 (37.5)	15 (62.5)	14 (58.3)	10 (41.7)	19 (79.2)	5 (20.8)	0.01^#^
OPRM1 rs1799971	AA	AG + GG	AA	AG + GG	AA	AG + GG	
*n (%)*	18 (75)	6 (25)	20 (83.3)	4 (16.7)	16 (66.7)	8 (33.3)	0.8
SCN9 A rs6746030	GG	GA + AA	GG	GA + AA	GG	GA + AA	
*n (%)*	13 (54.2)	11 (45.8)	20 (83.3)	4 (16.7)	18 (75)	6 (25)	0.7

*COMT* Catechol-O-Methyltransferase, *HTR1 A* 5-hydroxytryptamine receptor 1 A, *HTR1B* 5-hydroxytryptamine receptor 1B, *OPRM1* opioid receptor mu 1, *SCN9 A* sodium voltage-gated channel alpha subunit 9

Dwass-Steel-Critchlow-Fligner test *p < 0.05: Significant differences between TMD-R and the other groups, ^#^*p* < 0.05: Significant difference between the TMD-R and Control group

### Predictors of resistance

**Fig. 1:**
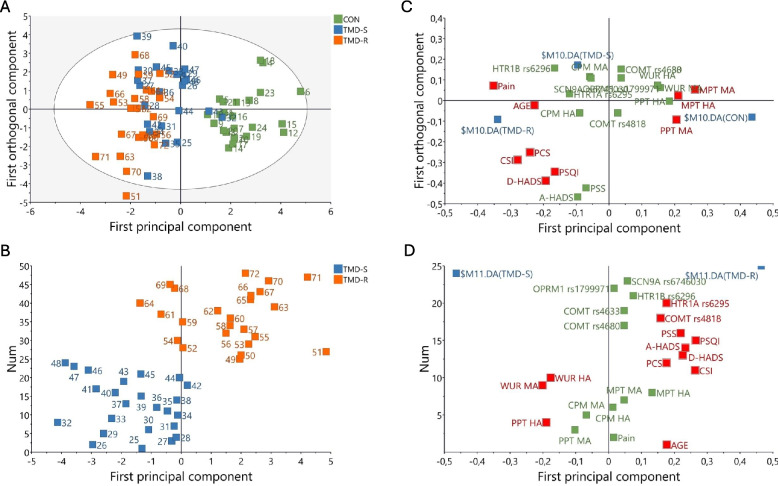
Orthogonal partial least square discriminant analysis (OPLS-DA) of patients with myogenous temporomandibular disorders (TMD) resistant to treatment (TMD-R), successful to treatment (TMD-S) and healthy control subjects (CON). **A** Score plots showing the separation between each observation in the TMD-R, TMD-S, and CON and **B** the separation between the TMD-R and TMD-S. **C** and **D** Loading plots showing the variables and their loadings with a VIP-value > 1.0 and p(corr) > 0.4 (red boxes). Green boxes are non-significant variables. Blue boxes represent group (diagnoses) separation. Significant variables correspond to those in Tables 4 and 5. PPT: pressure pain threshold; MPT: mechanical pain threshold; CPM: conditioned pain modulation; WUR: wind-up ratio; HADS: Hospital Anxiety (A) and Depression (D) Scale; CSI: Central Sensitization Inventory; PSS: Perceived Stress Scale; PSQI: Pittsburgh Sleep Quality Index; PCS: Pain Catastrophizing Scale, COMT: Catechol-O-Methyltransferase; HTR1 A: 5-hydroxytryptamine Receptor 1 A; HTR1B: 5-hydroxytryptamine receptor 1B; OPRM1: Opioid Receptor mu 1; SCN9 A: Sodium Voltage-Gated channel alpha subunit 9

The OPLS-DA showed one predictive component that was strongly significant (R2 = 0.453, Q2 = 0.418, CV-ANOVA *p*-value = 1.65e-12) (Fig. 1). The figure clearly shows the separation between the patients and controls, but also a separation between the TMD-R and TMD-S groups. There were nine variables that differed between the groups, i.e., had a VIP > 1.0 and a p(corr) > 0.4. Pain intensity was the strongest separator, followed by CSI and D-HADS. All intercorrelations p(corr) were negative and thus, lower in controls compared to the other groups (Table 4). As can be seen in the loading plot (Fig. 1) the psychosocial variables were more related to the TMD-R group, whereas the somatosensory variables were more related to the controls.

**Table 4:** OPLS-DA model of variables discriminating healthy controls from patients with temporomandibular disorders myofascial pain resistant to treatment (TMD-R) and with a successful treatment outcome (TMD-S). Nine variables discriminated between groups and had a VIP-value > 1.0 and a p(corr) > 0.4. Three variables (MPT Masseter and hand, PPT Masseter) had a positive p(corr) and were higher in controls, all other variables were lower in controls

	**VIP**	**p(corr)**
Pain intensity	1.76	−0.85
CSI	1.56	−0.67
D-HADS	1.37	−0.47
PCS	1.35	−0.58
MPT Masseter	1.31	0.63
PSQI	1.19	−0.40
Age	1.13	−0.55
MPT Hand	1.05	0.51
PPT Masseter	1.05	0.50

*CSI* Central Sensitization Index, *D-HADS* Hospital Anxiety and Depression Scale, depression subscale, *PCS* Pain Catastrophizing Scale, *MPT* mechanical pain threshold, *PSQI* Pittsburg Sleep Quality Index, *PPT* pressure pain threshold, *OPLS-DA* Orthogonal Partial Least Square Discriminant Analysis, *VIP* variable influence on projection; p(corr): correlation coefficient in multivariate analyses

As there was a separation also between the patient groups, we did a separate OPLS-DA to test which variables differed between TMD-R and TMD-S (Fig. 1). We found a highly significant predictive component (R2 = 0.564, Q2 = 0.456, CV-ANOVA *p*-value = 1.11e-6). Twelve of the variables had a VIP > 1 and p(corr) > 0.4 (Table 5). The three strongest separators between TMD-R and TMD-S were PSQI, CSI and D-HADS, but also some genetic variables, HTR1 A rs6295 and COMT rs4818 differed.

**Table 5:** OPLS-DA model of variables discriminating temporomandibular disorders myofascial pain patients resistant to treatment (TMD-R) from patients with a successful treatment outcome (TMD-S). Twelve variables significantly discriminated between groups and had a VIP-value > 1.0 and a p(corr) > 0.4. WUR and PPT had a negative p(corr) and were lower in TMD-R

	**VIP**	**p(corr)**
PSQI	1.69	0.76
CSI	1.67	0.76
D-HADS	1.49	0.67
A-HADS	1.44	0.65
PSS	1.40	0.63
WUR Masseter	1.28	−0.58
PPT Hand	1.21	−0.54
AGE	1.13	0.51
PCS	1.12	0.51
HTR1 A rs6295	1.12	0.51
WUR Hand	1.12	−0.50
COMT rs4818	1.01	0.45

*PSQI* Pittsburg Sleep Quality Index, *CSI* Central Sensitization Index, *D-HADS* Hospital Anxiety and Depression Scale, depression subscale, *A-HADS* anxiety subscale, *PSS* Perceived Stress Scale, *WUR* wind-up ratio, *PPT* pressure pain threshold, *PCS* Pain Catastrophizing Scale, *HTR1 A* serotonin type 1 receptor gene, *COMT* catecholamine-O-methyltransferase gene, *OPLS-DA* Orthogonal Partial Least Square Discriminant Analysis, *VIP* variable influence on projection; p(corr): correlation coefficient in multivariate analyses

## Discussion

To the best of our knowledge this is the first study phenotyping myofascial TMD patients considered as non-responders to conservative treatments. The main findings were 1) a low prevalence of resistance to treatment among myofascial TMD pain patients, 2) increased pain sensibility (PPT), increased pain amplification (WUR), higher levels of psychosocial distress, and a higher presence of the HTR1 A rs6295 polymorphism in the resistant TMD group compared with the other groups, and 3) that pain intensity, central sensitization, and depression were the strongest predictive variables for myofascial TMD pain, whereas sleep quality, central sensitization, depression, anxiety and stress were the strongest predictive variables for resistance to treatment, followed by somatosensory variables (WUR and PPT) and genetic variables (HTR1 A rs6295 and COMT rs4818).

Multifaceted treatment strategies in which reversible non-pharmacological therapies are considered the first option, followed by pharmacological options are recommended for TMD [37, 38]. Our resistant inclusion criteria followed these recommendations. Unsuccessful treatment with oral appliance, cognitive behavioral and pharmacotherapy (first with peripherally drugs and in case of lack of response centrally acting drugs were used), besides other treatments, were used as part of our criteria to classify myofascial TMD pain patients as resistant to treatments. Additionally, as the Initiative of Measurement, Methods, and Pain assessment in Clinical Trials (IMMPACT) recommendations consider treatments with a 30% pain reduction as effective [39, 40] and as this percentage is equivalent to “much improved” on the Patient Global Impression of Change Scale [41], we also included a pain reduction of less than 30% despite treatment as a criterion of resistance. Furthermore, the established three-month timeframe for unsuccessful treatment response, was based on responder definitions from other chronic pain conditions (such as fibromyalgia and low back pain) [42, 43], and on the resistant migraine criteria proposed by the European Headache Federation consensus [10], as these parameters have not yet been established for myogenous TMD patients. Validation of our criteria is necessary, as modifying any variable will undoubtedly impact the results, especially regarding the proposed time frame regarding resistance to treatment and the average pain intensity > 50. However, our criteria could be the first step in creating a responder definition that combines multiple significant outcomes into one metric, usable as a primary outcome in clinical trials. This unified metric could also guide clinical decision-making, replacing reliance on group averages for individual patient assessments [42].

Based on these criteria, our study found a low prevalence of resistant myofascial TMD pain patients. This finding was anticipated, primarily due to the low prevalence of TMD patients requiring treatment and the high response rate of this population to conservative treatments [13, 14]. However, since these patients were recruited from an orofacial pain specialist clinic, prevalence may be lower in general dentistry practices. This aligns reports on resistant migraines, with a higher prevalence (75%) in headache specialist centers than in primary care settings (13%) [11].

Regarding the somatosensory profile, patients of the TMD-R group presented a significant somatosensory gain of function (more sensitive), in the trigeminally and spinally innervated area, when WUR, and PPT were addressed. Taken together, these findings could indicate a generalized hypersensitivity of the central nervous system, inadequate functioning of the endogenous pain modulation system, and central sensitization [11, 44–46]. The lower PPT values found in the TMD-R group compared with the other groups and the non-significant differences between TMD-R and TMD-S for the MPT, could be more related to central hyperexcitability in both the trigeminal and spinal systems which are known to produce hyperalgesia, than to a higher proportion of sensitized primary nociceptive afferents [47]. Our multivariate analyses support this, demonstrating that lower MPT in both trigeminally and spinally innervated areas, as well as PPT in the trigeminal area, serve as predictors for myofascial TMD pain and lower PPT in the spinally innervated area is identified as a predictor of resistant myofascial TMD pain. In addition, myofascial TMD pain patients are more likely to present generalized mechanical hyperalgesia in the trigeminal and spinal regions than controls [48], which is in line with our findings. Additionally, a widespread lowering of PPT as showed in our study and in the literature, could reflect a deficit of the endogenous pain inhibitory mechanism [48, 49]. Our study demonstrated that the significant deficit in endogenous pain modulation observed in the TMD-R group was primarily due to increased pain facilitation [50], with a smaller contribution from descending pain inhibition, since the TMD-R group presented significant higher values in WUR and lower non- significant values in CPM compared with the other groups. These results supports findings from one study [51] but contradicts another [52]. It could be also questioned if the CPM was globally impaired among all TMD patients.

Painful TMD subjects often show moderate to severe somatization (28.5%−76.6%) and depression (21.4%−60.1%), and higher stress, catastrophizing, and hypervigilance compared to controls [53–57]. In line with these findings, our study revealed that the TMD-R group exhibited higher scores in all psychosocial assessments compared to the other groups; while the TMD-S group only showed worse outcomes for the CSI, PSQI and PCS compared with controls. It is important to note that studies have demonstrated that psychosocial factors (e.g. depression and stress), are significant predictors of new onset-TMD [58]. Longitudinal designs have also shown that these factors pose a risk for prolonged chronic pain in TMD patients [59–62]. This aligns with our multivariate analysis, which indicates that among the variables distinguishing patients from controls, four are related with psychosocial impairment (CSI, D-HADS, PCS and PSQI). Interestingly, among the 12 variables that differentiate resistant and non-resistant patients, the strongest predictors of belonging to the TMD-R group (PSQI, CSI, D/A-HADS and PSS) were also related with psychosocial impairment. These findings underscore the necessity of assessing psychosocial factors in TMD patients, as they appear to influence their response to treatment. Moreover, the Axis II of the short version of the DC/TMD (bDC/TMD) [63] can aid non-specialist clinicians in assessing psychosocial factors in a simpler and more interpretable manner. While psychological interventions such as cognitive behavioral therapy and counseling have shown a clinically relevant pain reducing effect in painful TMD patients [64], our findings highlight the need to include additional psychological treatments and to more effectively apply current treatments, given that counselling was used in our study. Also, our study reinforces the need of a multidisciplinary approach including psychologist specialized treatment. We recommend focusing on assessing central sensitization, depression, catastrophizing, and sleep quality, as these factors differentiated patients from controls and treatment responders from resistant patients.

The OPPERA study highlighted a genetic influence from single nucleotide polymorphisms (SNPs) in key biological pathways related to processing noxious stimuli and perceiving pain. These include genes related to serotonin receptor and catecho-O-methyltransferase (COMT) in relation to chronic TMD [65]. Our findings revealed that the TMD-R group showed a higher prevalence of the HTR1 A polymorphism (rs6295) compared to other groups, and the HTR1B polymorphism (rs6296) compared to the control group. This partially aligns with previous findings [65], as we assessed other serotonin receptor-related genes. Our study did not identify any genetic predictor for patients, which could be attributed to the small sample size. However, the HTR1 A rs6295 polymorphism and COMT rs4818 were found to be predictors for TMD-R patients. Serotonin, which can be considered both a neurotransmitter and a neuromodulator, is present in the peripheral and central nervous systems and affects pain amplification and inhibition within the serotonergic system [66, 67]. Clinical results suggest an increased peripheral release of serotonin in patients with chronic myalgia of the masseter and trapezius muscles [68, 69], and a relationship with allodynia of orofacial muscles [68]. However, a meta-analysis found no differences between local and generalized chronic musculoskeletal pain [70]. We can speculate that in our study serotonin may have affected peripheral and central sensitization in the TMD-R group. Similarly, studies have indicated that reductions in COMT activity can influence pain perception by indirectly regulating u-opioid receptor function [71] and decreasing the metabolism of epinephrine, thereby potentiating pain signaling through beta-adrenergic receptors [72, 73]. Thus, genetic polymorphisms that decrease COMT activity can contribute to the etiology of TMD and may have increased risk of developing persistent pain conditions like in our TMD-R group [74].

In summary, resistant myofascial TMD pain is not highly prevalent. The phenotype of these patients is primarily influenced by psychosocial impairment, followed by somatossensorial alterations, and distinctive genotype. Interestingly, the somatosensorial and psychosocial characteristics of the TMD-R group are similar to the “global symptoms cluster” identified in the OPPERA study, which exhibited increased pain sensitivity, higher psychological distress and widespread clinical symptoms, suggesting a higher prevalence of central sensitization [24]. The authors suggested that due to the impairments observed in pain processing, individuals in this cluster may be less responsive to treatments as in our study. Moreover, it is reasonable to consider that treatment resistance may be also influenced by factors related to the physician/dentist, particularly their knowledge of diagnosis and treatment [75]. Additionally, establishing the correct treatment plan from the initial appointment is crucial to prevent chronic pain [24]. Since the studied sample was treated by experienced orofacial pain specialists; these factors were minimized as much as possible. Furthermore, since compliance to the proposed treatments could have influenced the results, we recommend that future studies assess this variable in an objective manner, and not only on patient´s self-report. It is also recommended to assess demographic and social factors, medical comorbidities, sleep disorders, nutrition, sunshine and vitamin D, and microbiome as these have been identified as factors that may increase resistance to treatments [16]. Also, our results could not be extrapolated to TMD men populations, since we included solely women. Yet, labeling some as resistant, opens a window to irreversible therapies which is highly undesirable in non-lethal conditions as TMD. Finally, these findings hold potential for more individualized diagnosis and treatment.

## Conclusion

This is the first study presenting criteria to classify myofascial TMD pain patients as resistant to conventional treatments. Resistant myofascial TMD pain is not highly prevalent. Nevertheless, the phenotype of these patients includes psychosocial, somatosensory, and genetic factors, which may be considered risk factors for developing resistance to conservative myofascial TMD pain treatment. These results could help clinicians in identifying myofascial TMD pain patients resistant to treatments, in order to propose a more tailored treatment plan.

## Data Availability

No datasets were generated or analysed during the current study.
